# Hydrothermal Synthesis of Nanocomposites Combining Tungsten Trioxide and Zinc Oxide Nanosheet Arrays for Improved Photocatalytic Degradation of Organic Dye

**DOI:** 10.3390/nano15100772

**Published:** 2025-05-21

**Authors:** Chien-Yie Tsay, Tao-Ying Hsu, Gang-Juan Lee, Chin-Yi Chen, Yu-Cheng Chang, Jing-Heng Chen, Jerry J. Wu

**Affiliations:** 1 Department of Materials Science and Engineering, Feng Chia University, Taichung 407102, Taiwan; ms0228165@gmail.com (T.-Y.H.); chencyi@fcu.edu.tw (C.-Y.C.); yuchchang@fcu.edu.tw (Y.-C.C.); 2 Department of Academia-Industry Collaboration and Science Park Affairs, National Science and Technology Counil, Taipei 106214, Taiwan; gjlee@nstc.gov.tw; 3 Department of Photonics, Feng Chia University, Taichung 407102, Taiwan; jhchen@fcu.edu.tw; 4 Department of Environmental Engineering and Science, Feng Chia University, Taichung 407102, Taiwan; jjwu@fcu.edu.tw

**Keywords:** WO_3_/ZnO nanocomposite, heterojunction, hydrothermal method, visible-light photocatalyst, photoelectrochemical reaction, photodegradation

## Abstract

Both tungsten trioxide (WO_3_) nanosheet arrays and tungsten trioxide/zinc oxide (WO_3_/ZnO) nanocomposites were grown on fluorine-doped tin oxide (FTO) coated glass slides using a hydrothermal method to develop a visible-light-driven photocatalyst with easy reusability. Field emission scanning electron microscopy (FE-SEM) observations confirmed the formation of irregular oxide nanosheet arrays on the FTO surfaces. X-ray diffraction (XRD) analysis revealed the presence of hexagonal WO_3_ and wurtzite ZnO crystal phases. UV-Vis diffuse reflectance spectroscopy showed that integrating ZnO nanostructures with WO_3_ nanosheets resulted in a blue shift of the absorption edge and a reduced absorption capacity in the visible-light region. Photoluminescence (PL) spectra indicated that the WO 0.5/ZnO 2.0 sample exhibited the lowest electron-hole recombination rate among the WO_3_/ZnO nanocomposite sample. Photocatalytic degradation tests demonstrated that all WO_3_/ZnO nanocomposite samples had higher photodegradation rates for a 10 ppm methylene blue (MB) aqueous solution under visible-light irradiation compared to pristine WO_3_ nanosheet arrays. Among them, the WO 0.5/ZnO 2.0 sample showed the highest photocatalytic efficiency. Furthermore, it exhibited excellent recyclability and high photodegradation stability over three cycles.

## 1. Introduction

Due to rapid advancements in emerging technologies and the globalization of economic and trade activities, global industrial output has grown significantly. As a result, countries around the world face challenges such as energy shortages and environmental pollution [1]. Among various solution approaches, nanoscale oxide semiconductor photocatalytic materials have attracted considerable attention [2,3]. These materials can absorb solar energy and induce photoelectrochemical reactions to degrade organic contaminants and pollutants, making them a key technology for addressing energy shortages and promoting environmental sustainability [4,5]. The development and applications of such material systems have gained widespread interest.

The optical bandgap energy, microstructural morphology, specific surface area, and the separation and recombination characteristics of photogenerated charge carriers are critical factors influencing the photocatalytic performance of oxide semiconducting materials [6]. These properties play key roles in the selection of practical and promising oxide photocatalysts. In addition to physical characteristics, factors such as cost efficiency, environmental friendliness, and reusability are also important considerations when selecting suitable material systems. Semiconductor photocatalytic nanomaterials, such as TiO_2_, ZnO, WO_2_, MnO_2_, Mo_2_O_3_, CuO, Cu_2_O, and SnO_2_, have been widely utilized for the photodegradation of organic contaminants by harnessing solar energy to initiate photoelectrochemical reactions [7,8]. Among these contaminants, organic dyes are particularly significant, representing a major source of industrial wastewater pollution, and pose serious environmental threats by severely contaminating rivers and the surrounding soil.

Several strategies have been used to enhance the photocatalytic activity of semiconductor photocatalysts by improving the generation efficiency of photocarriers, suppressing the recombination of electron-hole pairs, and increasing the mobility of charge carriers [2]. These approaches include improving crystal quality, inducing phase transitions, modifying chemical composition through impurity doping, controlling the microstructure, regulating the bandgap, applying surface sensitization, and coupling with other semiconductors to form heterojunction composites [9].

Zinc oxide (ZnO) is an n-type semiconductor and a promising photocatalyst candidate due to its superior photocatalytic degradation performance in organic compounds compared to typical titanium dioxide (TiO_2_) [8]. ZnO has a wide bandgap energy of approximately 3.35 eV, enabling the excitation of photoinduced charge carriers under near-UV light irradiation. However, it suffers from a high recombination rate of photogenerated electron-hole pairs, which could reduce its photocatalytic efficiency and limit its application in photoelectrochemical processes [10,11]. Tungsten trioxide (WO_3_) is also an n-type semiconductor, exhibiting excellent photoelectrochemical properties, and has high chemical stability [12]. With a narrower optical bandgap ranging from 2.4 to 2.8 eV, WO_3_ can absorb visible light to improve optoelectronic properties, making it more effective in utilizing solar energy for photoelectrochemical applications [13]. Numerous previous studies demonstrated that the combination of ZnO and WO_3_ to form heterostructure nanocomposites not only improves visible-light absorption but also enhances the separation of photogenerated charge carriers while suppressing their recombination [14,15,16]. Therefore, this feature leads to a significant improvement in photocatalytic degradation efficiency under visible-light irradiation.

For example, Lam et al. investigated various loadings of WO_3_ nanoparticles coated with ZnO nanorods, synthesized using a combination of hydrothermal and chemical solution processes, for applications in photocatalytic degradation [17]. Their study demonstrated that WO_3_–ZnO nanocomposites exhibited higher photocatalytic activity compared to pure ZnO nanorods and commercial P25. This enhancement was attributed to the formation of a heterostructure that promotes the separation of photoinduced charge carriers and improves the surface properties of the oxide semiconductor photocatalyst. Zheng et al. fabricated composite structures consisting of WO_3_ nanorod arrays and ZnO nanosheet arrays on FTO substrates [14]. They investigated the influence of the ZnO hydrothermal growth time on the physical properties and methylene blue (MB) photodegradation performance. Their results showed that the WO_3_/ZnO composite structures exhibited significantly better photodegradation efficiency compared to individual WO_3_ nanorod arrays and ZnO nanosheet arrays. Furthermore, Lei et al. reported the fabrication of ZnO nanoparticles deposited on WO_3_ nanosheet arrays, which were grown on a 316 L stainless steel substrate through a hydrothermal process followed by thermal treatment, for the photocatalytic degradation of the organic dye methylene blue (MB) under solar light irradiation [18]. They investigated the effects of hydrothermal reaction time (1–5 h) at 120 °C for WO_3_ nanosheet arrays (NSAs) and varying concentrations of ZnO precursor solution (10–50 mM, in 10 mM increments) on the structural and photochemical properties of WO_3_/ZnO NSAs. The results showed that the WO_3_/ZnO heterojunction, when loaded with an optimal amount of ZnO nanoparticles, exhibited significantly enhanced photocatalytic activity compared to the pristine WO_3_ nanosheet arrays. This improvement was attributed to the favorable energy band alignment of the heterojunction, enhanced light absorption, and efficient separation of photogenerated electron-hole pairs, all of which contribute to the improved photocatalytic performance and stability.

Typical nanopowder photocatalysts require inconvenient separation and drying for reuse, often leading to material loss. Depositing nanostructured photocatalysts on FTO glass substrates offers a more convenient and reusable alternative. Furthermore, the FTO bottom layer improves light absorption and improves photocatalytic performance. In the present study, the authors prepared both WO_3_ nanosheet arrays and WO_3_/ZnO nanocomposites on transparent conducting FTO glass substrates using a low-temperature hydrothermal method. The aim is to develop photocatalysts with high degradation efficiency under visible light and easy reusability potential. The effects of hydrothermal growth time on the structural properties, optical characteristics, and organic dye photodegradation efficiency of the WO_3_ nanosheet arrays and WO_3_/ZnO nanocomposites were systematically investigated. Additionally, the oxide semiconductor photocatalyst that exhibited the highest degradation performance was subjected to a cycling test to evaluate its reusability.

## 2. Materials and Methods

### 2.1. Synthesis Procedures of WO_3_ Nanosheet Arrays and WO_3_/ZnO Nanocomposites

Before growing WO_3_ nanosheet arrays and WO_3_/ZnO nanocomposites on FTO glass (20 × 10 × 2 mm^3^, TECA7 (6–8 Ohm/□), Pilkington, Lathom, UK), the substrates were cleaned using ultrasonic vibration, blown dry with nitrogen, and then dried on a hot plate. To prepare a 0.1 M WO_3_ hydrothermal reaction solution, sodium tungstate dihydrate (Na_2_WO_4_·2H_2_O, 99%, Alfa Aesar, Haverhill, MA, USA) was first dissolved in 25 mL of deionized (DI) water. A measured amount of hydrochloric acid (HCl, 2 M, 37%, Honeywell, Charlotte, NC, USA) was then added under magnetic stirring to acidify the solution. The mixture was diluted to a final volume of 125 mL and the pH was adjusted to 2.0, resulting in the formation of a white precipitate [14,15]. After stirring for 30 min, sodium chloride (NaCl, 99.5%, SHOWA, Gyoda, Japan) was added to the solution. The resulting precursor solution was transferred to a Teflon-lined stainless steel autoclave. Then, an FTO glass substrate was fully immersed in the WO_3_ precursor solution at an angle of approximately 45° during the hydrothermal reaction. The sealed autoclave was placed in a box oven and maintained at 170 °C for 0.5, 1.0, or 2.0 h, respectively. After the reaction, the autoclave was allowed to cool naturally to room temperature. The hydrothermally synthesized sample was then removed, rinsed thoroughly with DI water, and dried at 70 °C in air.

To synthesize the WO_3_/ZnO nanocomposites, the as-prepared WO_3_ nanosheet array sample was immersed in a ZnO precursor solution, again at a 45° tilt during the hydrothermal process. The 0.1 M ZnO precursor solution was prepared by mixing 40 mL of 0.1 M zinc nitrate (Zn(NO_3_)_2_·6H_2_O, 99%, Alfa Aesar) solution with an equal volume of 0.1 M hexamethylenetetramine (HMT, C_6_H_12_N_4_, 99%, SHOWA) solution, followed by magnetic stirring for 30 min. During the hydrothermal reaction, the sealed autoclave was placed in a box oven and maintained at 95 °C for 2 h [18]. After the reaction, the WO_3_/ZnO nanocomposite samples were removed, washed with DI water, and dried for subsequent physical characterization and photocatalytic degradation experiments. All chemicals used in this study were analytical grade.

### 2.2. Physical Properties Characteristics and Photocatalytic Degradation Performance Measurement

The crystal structure of the as-synthesized oxide nanostructures was examined using an X-ray diffractometer (Bruker D8 Discover, Bruker, Billerica, MA, USA) with Cu Kα radiation, scanning in the 2θ range of 10° to 80°. Surface morphology was characterized using a Hitachi S-4800 scanning electron microscope (Hitachi High-Technology, Tokyo, Japan, SEM), equipped with an energy-dispersive X-ray spectroscopy (EDS) detector for elemental analysis. The Raman spectra of the oxide nanostructures were recorded in the spectral range of 200 to 1200 cm^−1^ using a HORIBA Scientific LabRAM HR Evolution Raman system (HORIBA Jobin Yvon, Paris, France) with an Ar laser source at an excitation wavelength of 532 nm. UV-Vis diffuse reflectance spectra (DRS), in the wavelength range of 300–800 nm, were measured using a PERKIN ELMER LAMBDA 650 UV/V spectrophotometer (Shelton, CT, USA) to analyze optical absorption properties and estimate the optical bandgap. Photoluminescence (PL) measurements were performed on a SHIMADZU RF-5301PC spectrophotometer (Kyoto, Japan) using a UV laser excitation wavelength of 325 nm.

The photocatalytic activities of the WO_3_ nanosheet array samples and the WO_3_/ZnO nanocomposite samples were evaluated by measuring the degradation rate of a dilute solution of methylene blue (MB) under visible-light irradiation for different times. Each photocatalyst sample was immersed in 100 mL of an aqueous MB solution with a concentration of 10 mg/L. To achieve adsorption equilibrium between the photocatalyst sample and the dye molecules, the solution was stirred in the dark for 60 min prior to illumination. Subsequently, the solution was irradiated with a 300 W visible-light source from a xenon (Xe) lamp (total power 500 W, λ ≥ 420 nm), positioned approximately 60 cm above the surface of the solution. At predetermined time intervals, aliquots of the solution were collected, and the concentration of MB was determined by measuring its absorbance using a Hitachi U-2900 double beam spectrophotometer (Hitachi High-Technology, Tokyo, Japan).

## 3. Results and Discussion

**Figure 1:**
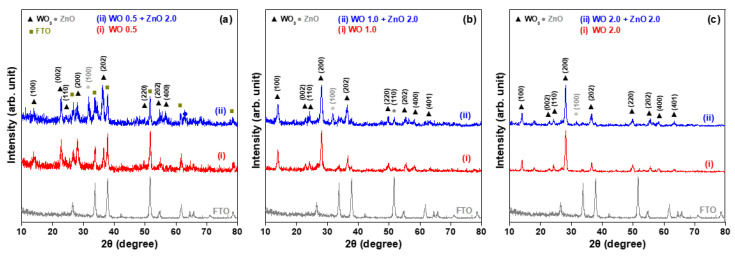
X-ray diffraction (XRD) patterns of hydrothermally synthesized WO_3_ nanosheet arrays and WO_3_/ZnO nanocomposites on FTO glass substrates. The WO_3_ growth times are (**a**) 0.5 h, (**b**) 1.0 h, and (**c**) 2.0 h.

Figure 1 shows the XRD patterns of WO_3_ nanosheet arrays grown on FTO glass substrates using a hydrothermal method at 170 °C for 0.5, 1, and 2 h. The eight X-ray diffraction peaks correspond to the crystal planes (100), (002), (110), (200), (202), (220), (222), and (400) of WO_3_, as indexed in JCPDS card No. 33-1378 [15]. These results confirm that the hydrothermally synthesized WO_3_ nanostructures are hexagonal in nature. In addition to detecting signals from the WO_3_ crystal phase, diffraction peaks from the FTO film are also detected. This is because, with a short growth duration of 0.5 h, the thickness of the WO_3_ nanosheet array is less than the penetration depth of the Cu Kα radiation used in XRD examination, allowing the diffraction signals of the underlying FTO film to be observed. These XRD patterns also indicate that as the growth time increases, the intensity of the diffraction peaks becomes stronger and the peak shapes sharper. This suggests not only an increase in the thickness of the WO_3_ nanosheet arrays, but also an improvement in their crystallinity.

Figure 1 also presents the XRD patterns of WO_3_/ZnO nanocomposites, which were prepared by first growing WO_3_ nanosheet arrays on FTO glass substrates by hydrothermal synthesis with varying durations, followed by a second hydrothermal step to deposit ZnO nanostructures on the WO_3_ arrays. The three WO_3_/ZnO samples show diffraction peaks corresponding to the crystalline phases of both WO_3_ and ZnO. These peaks align well with standard JCPDS No. 33-1378 for the WO_3_ phase and No. 36-1451 for the ZnO phase [15], confirming the successful formation of WO_3_/ZnO heterostructured nanocomposites. For the WO 0.5 + ZnO 2.0 sample (pattern (ii) of Figure 1a), the XRD pattern reveals diffraction signals from three distinct phases: FTO, WO_3_, and ZnO. In particular, the diffraction peaks (002) and (200) of WO_3_ are prominent. As the hydrothermal growth time of the WO_3_ nanosheets increases, the intensity of the (002) peak decreases significantly, while the (200) peak becomes dominant. This trend suggests a gradual shift in the preferred growth orientation of the WO_3_ nanosheets from the [002] to the [200] direction. The hexagonal phase of the WO 2.0 + ZnO 2.0 sample is well-matched to the 2θ positions and relative intensities listed on JCPDS card No. 33-1378 (pattern (ii) of Figure 1c). Furthermore, the intensity of ZnO-related XRD peaks, specifically (100) and (110), decreases with increasing WO_3_ growth time. Since the duration of ZnO growth remained constant for all samples, the strongest ZnO diffraction peaks appear in the sample where the WO_3_ nanosheets were grown for only 0.5 h. As the thickness and size of the WO_3_ nanosheets increase, the corresponding ZnO XRD peaks become weaker, indicating a reduced contribution of the ZnO phase to the overall structure.

**Figure 2:**
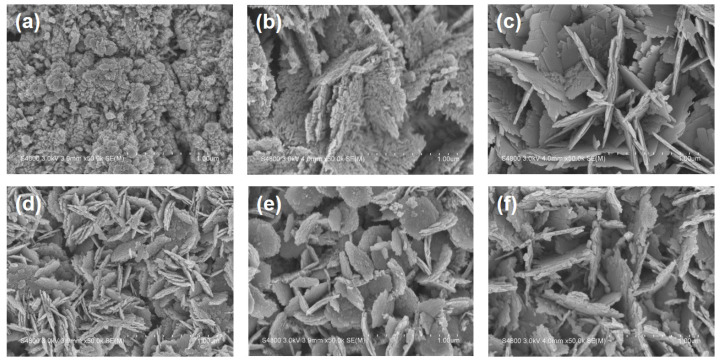
Plan-view field-emission scanning electron microscopy (FE-SEM) micrographs of (**a**–**c**) WO_3_ nanosheet arrays and (**d**–**f**) WO_3_/ZnO nanocomposites deposited on FTO glass substrates. WO_3_ was grown for 0.5 h (**a**,**d**), 1.0 h (**b**,**e**), and 2.0 h (**c**,**f**), respectively. For the nanocomposites (**d**–**f**), ZnO was deposited with a fixed growth time of 2.0 h following WO_3_ growth.

In the present study, WO_3_ nanostructures were grown directly on FTO glass substrates, using the polycrystalline nature of FTO as a temporary seed layer. Figure 2a–c presents top-view FE-SEM micrographs of WO_3_ nanosheet arrays synthesized at various durations of the hydrothermal reaction. After 0.5 h of growth (Figure 2a), only irregularly shaped WO_3_ nanocrystalline particles were observed on the surface. When the hydrothermal reaction time was extended to 1 h (Figure 2b), these initial nanocrystalline particles, formed during the early stage of growth, acted as nucleation sites for the development of nanosheet structures. This FE-SEM image shows that each nanosheet exhibits a rough and porous surface morphology, which is advantageous for enhancing light absorption and providing active sites for photocatalytic reactions. Furthermore, in the absence of a pre-deposited WO_3_ seed layer, the growth orientation of the nanosheet arrays was not aligned vertically with the substrate, but rather occurred in random and irregular directions. When the reaction time increased further to 2 h (Figure 2c), the WO_3_ nanosheets became larger and thicker, and their surfaces became smoother and denser. The thickness of WO_3_ nanosheets grown for different hydrothermal durations was estimated using cross-sectional FE-SEM micrographs. The average thicknesses of the W 0.5, W 1.0, and W 2.0 samples were determined to be approximately 525 nm, 890 nm, and 1050 nm, respectively.

Figure 2d–f shows FE-SEM micrographs of WO_3_/ZnO heterostructure composites synthesized with different hydrothermal growth of the WO_3_ nanostructures. As the growth time increases, the FE-SEM images reveal that the nanosheets in the WO_3_/ZnO heterostructures become progressively larger and thicker. The estimated average thickness and length of the WO_3_/ZnO nanoflakes are approximately 36 and 357 nm, 72 and 536 nm, and 89 and 895 nm for the composites WO 0.5 + ZnO 2.0, WO 1.0 + ZnO 2.0, and WO 2.0 + ZnO 2.0, respectively. These values were obtained from the corresponding FE-SEM micrographs of WO_3_/ZnO nanoflake samples. Moreover, the surfaces of these nanosheets are not smooth, but decorated with numerous nanoparticles, forming a hierarchical structure. This hierarchical architecture effectively increases the specific surface area and provides a greater number of active sites for photocatalytic reactions. Compared to flat nanosheet surfaces, the rough morphology composed of surface-bound nanoparticles enhances the absorption of incident light energy. Furthermore, compared to the WO_3_ nanosheet arrays shown in Figure 2a–c, the WO_3_/ZnO nanocomposite exhibits more densely packed nanosheet structures. This compact heterostructure is anticipated to improve photoelectrochemical performance, thereby enhancing the efficiency of photocatalytic degradation.

**Figure 3:**
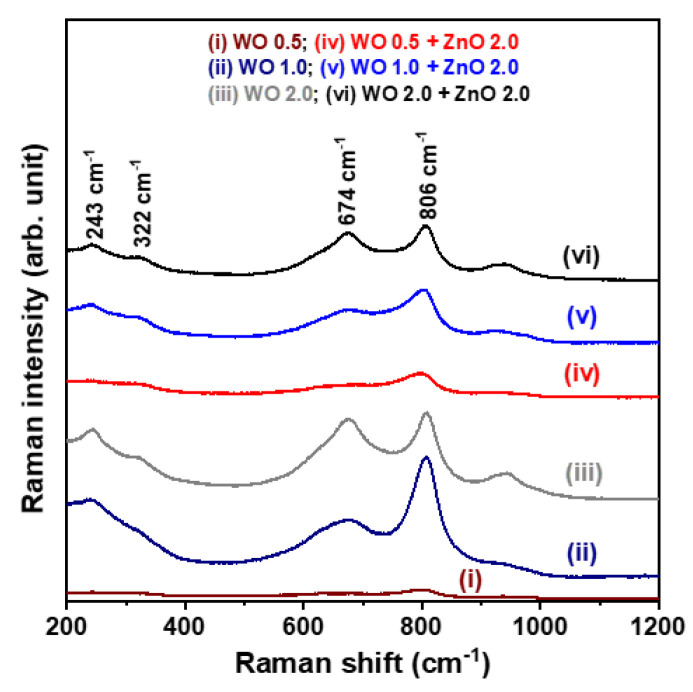
Raman spectra of WO_3_ nanosheet array samples and WO_3_/ZnO nanocomposite samples.

Figure 3 presents the Raman spectra of the WO_3_ nanosheet arrays and the WO_3_/ZnO nanocomposites, measured at room temperature in the range of 200–1200 cm^−1^. As shown in the figure, characteristic Raman signals appear at 674 and 809 cm^−1^, corresponding to the stretching and bending vibrations of W–O–W bonds in the WO_3_ nanocrystals [19]. However, no Raman peaks associated with ZnO nanocrystals are detected in the spectra of the WO_3_/ZnO nanocomposite samples. Despite this, the previous XRD analysis confirmed the presence of the ZnO phase by identifying diffraction peaks corresponding to the (100) and (110) planes. Furthermore, the SEM-EDS spectra confirm the presence of W, O, and Zn elements in the synthesized WO_3_/ZnO nanocomposites, verifying the successful incorporation of ZnO into the composite structure. The absence of ZnO-related Raman signals is likely due to the relatively low ZnO concentration in the WO_3_/ZnO nanocomposites. This interpretation is supported by the XRD results, where the ZnO (100) and (110) diffraction peaks are much weaker than those of the WO_3_ phase, suggesting that the ZnO content is insufficient to generate detectable Raman signals under the measurement conditions used.

**Figure 4:**
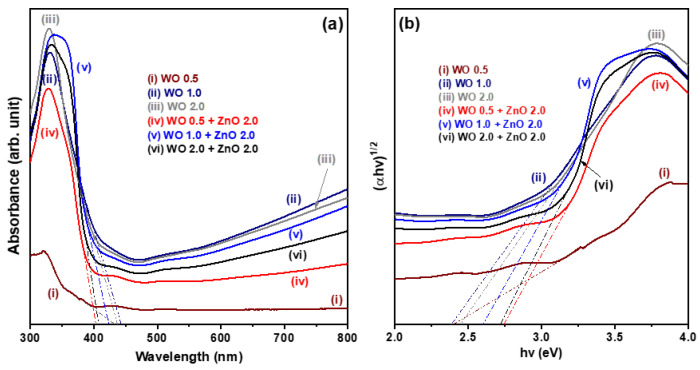
(**a**) UV-Vis diffuse reflectance absorption spectra and (**b**) the corresponding Tauc plots for WO_3_ nanosheet array samples and WO_3_/ZnO nanocomposite samples.

Figure 4a shows the UV-Vis absorption spectra of both pure WO_3_ nanosheet array samples and WO_3_/ZnO nanocomposite samples. The absorption edges of the three pure WO_3_ nanosheet samples lie within the range of 430–445 nm. This characteristic absorption corresponds to electronic transitions from the valence band to the conduction band of the WO_3_ nanosheets, indicating its optical bandgap energy. In comparison, the WO_3_/ZnO nanocomposite samples exhibit absorption edges between 400–425 nm. This noticeable blue shift relative to the pure WO_3_ samples is attributed to the influence of the ZnO nanostructures. Additionally, the WO 1.0 and WO 2.0 samples show stronger visible-light absorption than the three WO_3_/ZnO nanocomposites. However, as the hydrothermal reaction time increases, the visible-light absorption of the WO_3_/ZnO nanocomposites improves significantly. This enhancement is likely due to the increased thickness of the WO_3_ nanosheet arrays in the WO_3_/ZnO nanocomposites, which enhances the light-harvesting capability in the visible region.

Figure 4b displays the corresponding Tauc plots, illustrating the relationship between (αhν)^1/2^ and the photon energy (hν), based on the UV-Vis diffuse reflectance data recorded. The optical bandgap energy (Eg) of the oxide nanostructures is estimated using the following Tauc equation:

(αhν)^1/2^ = A(hν − Eg), (1)

where A is a constant, h is Planck’s constant, ν is the frequency of incident light, α is the absorption coefficient, and Eg is the optical bandgap energy of the oxide semiconductor. By extrapolating the linear portion of each curve in Figure 4b to the *X*-axis (photon energy), the Eg values were determined. The estimated optical bandgap energies for the six oxide nanostructure samples are approximately 2.40, 2.40, 2.44, 2.77, 2.63, and 2.71 eV, respectively.

**Figure 5:**
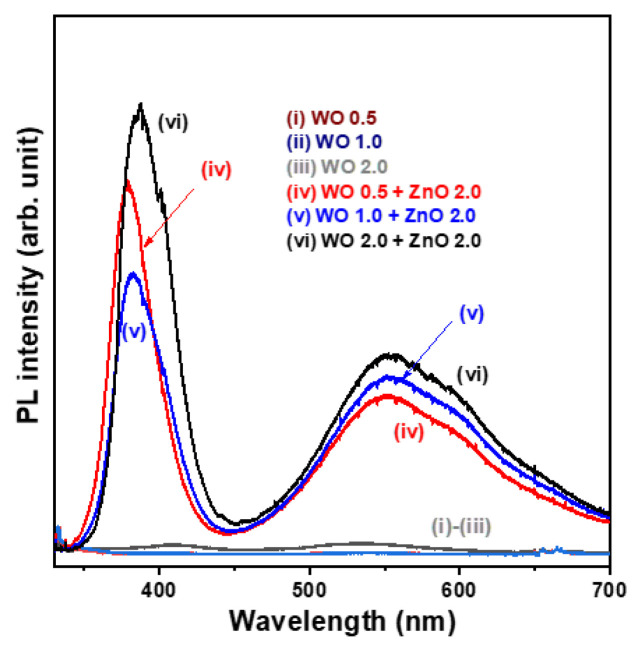
Comparison of photoluminescence (PL) spectra for three WO_3_ nanosheet array samples and three WO_3_/ZnO nanocomposite samples.

The photoluminescence (PL) spectra of the nanostructural oxide samples measured at room temperature are shown in Figure 5. The WO_3_ nanosheet array samples exhibit very weak PL signals (spectra (i)–(iii)). On the contrary, all WO_3_/ZnO nanocomposite samples display two prominent emission peaks, including a near-ultraviolet emission peak at approximately 355.4 nm, attributed to near-band edge transitions, and a broad, intense visible emission band ranging from 450 to 650 nm, which is generally associated with crystal defects (spectra (iv)–(vi)). The significantly enhanced PL emission intensity observed in the WO_3_/ZnO heterostructure samples can be attributed to the direct optical bandgap nature of ZnO, which facilitates the generation of electron-hole pairs upon photoexcitation. The broad visible emission band in the 450–650 nm range (yellow-green light region) is likely due to oxygen vacancies and tungsten interstitials. As the growth time of the WO_3_ nanosheet arrays in the WO_3_/ZnO nanocomposite samples increased from 0.5 to 2.0 h, the near-UV PL emission peak exhibited a slight redshift. Furthermore, the intensity of the defect-related visible emission band increased markedly, indicating a higher rate of photogenerated electron-hole pairs with an increased WO_3_ content. This suggests that the WO 0.5 + ZnO 2.0 sample exhibits (spectrum (iv)) the lowest electron-hole recombination rate among the tested samples.

**Figure 6:**
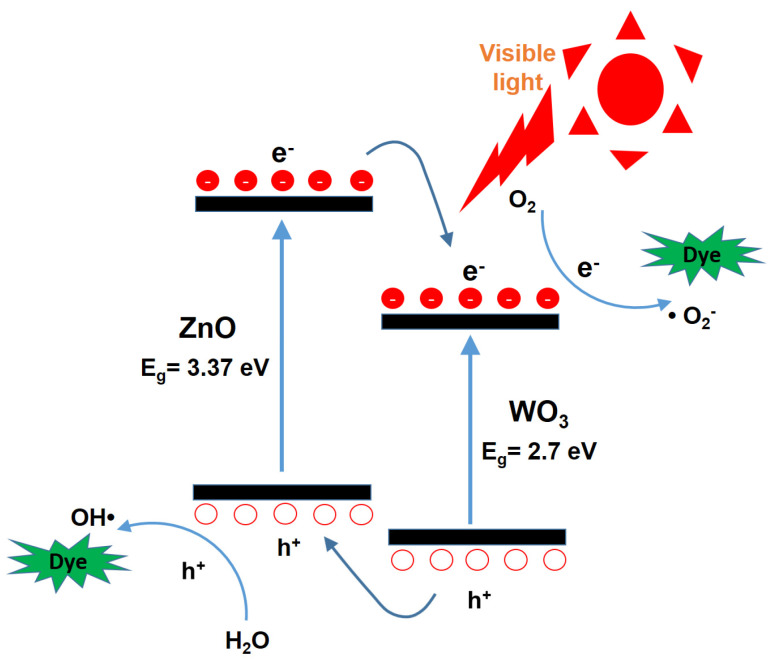
The energy band diagram and possible photocatalytic degradation mechanisms in the WO_3_ and ZnO system.

Figure 6 presents a schematic energy band diagram of the ZnO/WO_3_ heterojunction, illustrating the charge transfer process and the reactions of photogenerated electrons and holes with surface-adsorbed oxygen and water molecules on the nanocatalysts, respectively. It is well-known that photogenerated electrons in the conduction band (CB) of ZnO can rapidly transfer to the CB of WO_3_, while the corresponding photogenerated holes in the valence band (VB) of WO_3_ migrate to the VB of ZnO due to the favorable band alignment of the heterojunction [8,15]. Electrons react with oxygen molecules adsorbed on the nanocatalyst surface to generate superoxide anion radicals (•O_2_^−^), while holes oxidize water molecules to produce hydroxyl radicals (•OH) [20]. These reactive oxygen species possess strong oxidative capabilities, enabling the degradation of organic dyes into smaller, nontoxic molecules such as carbon dioxide and water [21]. This charge separation and reactive radical generation mechanism is considered a key factor contributing to the enhanced photocatalytic performance of heterojunction-based composite photocatalysts.

**Figure 7:**
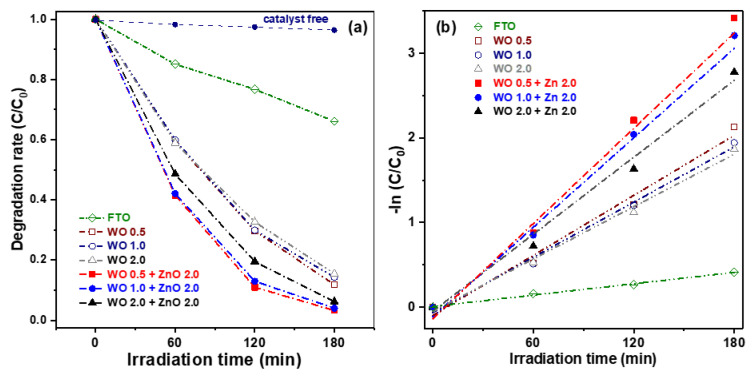
(**a**) Photocatalytic degradation rates under visible-light irradiation as a function of illumination time and (**b**) corresponding first-order kinetic plots for FTO glass, three WO_3_ nanosheet photocatalysts, and three WO_3_/ZnO nanocomposite photocatalysts.

Wastewater from industries such as textiles, food processing, papermaking, printing, leather, and cosmetics often contains azo dyes. The direct discharge of such untreated wastewater into rivers poses a serious threat to aquatic ecosystems and human health, due to the potential for biological mutations and carcinogenic effects. Consequently, photocatalytic degradation studies frequently employ model pollutants such as methylene blue (MB), methyl orange (MO), and rhodamine B (RhB) [22]. Figure 7a illustrates the degradation rate of aqueous MB solutions under visible-light irradiation using WO_3_ nanosheets and WO_3_/ZnO nanocomposites, plotted as a function of the irradiation time. To evaluate the effect of photocatalysts on the degradation of MB dye, an experiment without any photocatalyst was performed. In the absence of a photocatalyst, the degradation rate of MB dye was negligible, reaching only about 3.6% after 180 min of illumination. For comparison, the photocatalytic performance of FTO glass was also measured as a reference. After 180 min of visible-light illumination, the FTO glass exhibited a degradation rate of only approximately 20%. The three pure WO_3_ nanosheet samples showed similar photocatalytic performance, with degradation efficiencies ranging from 84.5% to 86.0%, indicating minimal variation among them. In contrast, the WO_3_/ZnO nanocomposite samples exhibited significantly enhanced degradation efficiency compared to the pure WO_3_ samples. This improvement can be attributed to the higher specific surface area of the nanocomposites, which provides more active sites during the photoelectrochemical reactions. Additionally, the formation of WO_3_/ZnO heterojunctions effectively suppresses the recombination of photogenerated electron-hole pairs, a key factor contributing to improved photocatalytic activity. Among those oxide semiconductor photocatalysts, the WO 0.5 + ZnO 2.0 nanocomposite exhibited the highest photocatalytic performance, achieving an MB degradation efficiency of 96.7% after 180 min of visible-light irradiation.

Figure 7b presents the photocatalytic kinetics of the six samples, plotted as –ln(C/C_0_) versus illumination time. The data were fitted using a pseudo-first-order kinetic model, described by the following equation:

−ln(C/C_0_)^1/2^ = *K*t, (2)

where *K* is the reaction rate constant, *C*_0_ is the initial concentration of MB, *C* is the concentration of MB at time *t* (in minutes), and *t* is the irradiation time. The calculated *K* values for the three pure WO_3_ samples were 0.0118, 0.0109, and 0.0103 min^−1^, respectively. Meanwhile, the K values for the WO_3_/ZnO nanocomposites were significantly higher, at 0.0193, 0.0182, and 0.0154 min^−1^. In particular, the WO 0.5 + ZnO 2.0 sample exhibited the highest rate constant, representing an approximate 63.6% increase compared to the WO 0.5 sample alone. Based on these results, the WO 0.5 + ZnO 2.0 nanocomposite was selected for photocatalytic repeatability tests due to its superior degradation performance.

**Figure 8:**
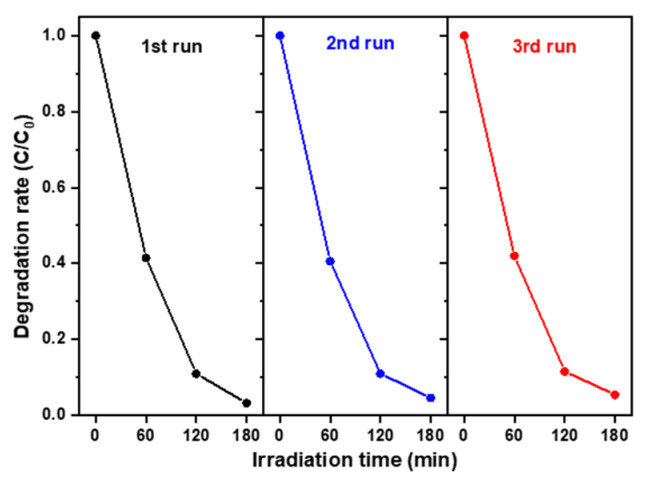
Recycling photodegradation characteristics of the WO_3_/ZnO nanocomposite photocatalyst, with WO_3_ grown for 0.5 h and ZnO for 2.0 h, evaluated for degradation of 10 ppm MB aqueous solution under visible-light irradiation.

Table 1 summarizes a comparison of key experimental parameters and results between this study and several published articles, highlighting the relatively low dye concentration used in this work and the achievement of the highest degradation rate after 3 h of photocatalytic treatment. Investigating the reusability of the developed oxide photocatalyst is a key focus of this study. Figure 8 shows the photocatalytic degradation rate of the WO 0.5 + Zn 2.0 sample under visible-light irradiation over multiple cycles of use to degrade MB in an aqueous solution. After each photodegradation test, the sample was rinsed with DI water, dried on a hot plate at 50 °C for 10 min, and then reused. After two reuse cycles, the degradation efficiency showed a slight decrease from 96.7% to 93.4%, corresponding to a reduction of approximately 3.3%. This minor reduction in performance may be due to residual organic dye remaining on the photocatalyst surfaces or to the partial detachment of the oxide nanocomposite film during use. Therefore, enhancing the adhesion characteristics between the oxide nanocomposite film and the FTO layer is essential before this material can be advanced toward practical engineering applications.

## 4. Conclusions

WO_3_/ZnO nanocomposites with varying thicknesses of WO_3_ nanosheet arrays were grown on FTO glass using the hydrothermal method. Integration of WO_3_ nanosheets with ZnO nanostructures reduced visible-light absorption and resulted in a wider optical bandgap energy compared to the WO_3_ nanosheet array samples. Photoluminescence (PL) emission spectra indicated that the WO 0.5/ZnO 2.0 sample exhibited a lower electron-hole pair recombination rate than the other WO_3_/ZnO nanocomposites. Overall, the WO_3_/ZnO nanocomposite photocatalysts demonstrated better photocatalytic activity compared to pristine WO_3_ nanosheet arrays under visible illumination, and the WO 0.5/ZnO 2.0 sample achieved the highest photodegradation efficiency in this study. Furthermore, depositing WO_3_/ZnO nanocomposites onto FTO glass supports easy separation from the treated aqueous solution and allows reuse in the photocatalytic degradation of organic dyes. The WO 0.5/ZnO 2.0 nanocomposite sample retains approximately 96.6% photocatalytic efficiency after two consecutive recycling cycles. It shows potential for practical application in wastewater treatment.

## Figures and Tables

**Table 1:** Compares the fabrication processes, material characteristics, and photodegradation performance of this work with several previous studies.

Catalyst	Material Form or Substrate	Fabrication Process	Dye Concentration	Degradation Time (h)	Degradation Efficiency (%)	Ref. No.
WO_3_/ZnO	Powder (0.05 g)	Hydrothermal method and combustion method	MB (10 ppm, 50 mL)	2	93	16
WO_3_/ZnO	Stainless steel 3.5 × 1.0 cm^2^	Hydrothermal method and dip coating	MB (5 ppm, 20 mL)	1.5	90	18
WO_3_/ZnO	FTO glass 3.0 × 2.0 cm^2^	Hydrothermal method	MB (16 ppm, 200 mL)	1	80	14
WO_3_/ZnO	FTO glass 2.0 ×1.0 cm^2^	Hydrothermal method	MB (10 ppm, 100 mL)	3	96.6	This work

## Data Availability

The experimental results and the corresponding data are presented in this article.
